# Characterization of Catalase from Psychrotolerant *Psychrobacter piscatorii* T-3 Exhibiting High Catalase Activity

**DOI:** 10.3390/ijms13021733

**Published:** 2012-02-07

**Authors:** Hideyuki Kimoto, Kazuaki Yoshimune, Hidetoshi Matsuyma, Isao Yumoto

**Affiliations:** 1 Department of Bioscience and Technology, School of Engineering, Tokai University, Minamisawa, Minami-ku, Sapporo 005-8601 Japan; E-Mails: hideyuki-kimoto3587@hotmail.co.jp (H.K.); matsuyama@tspirit.tokai-u.jp (H.M.); 2 Bioproduction Research Institute, National Institute of Advanced Industrial Science and Technology (AIST), 2-17-2-1 Tsukisamu-Higashi, Toyohira-ku, Sapporo, 062-8517 Japan; 3 Department of Applied Molecular Chemistry, College of Industrial Technology, Nihon University, 1-2-1, Izumichou, Narashino, Chiba 275-8575 Japan; E-Mail: yoshimune.kazuaki@nihon-u.ac.jp

**Keywords:** catalase, *Psycrobacter piscatorii*, PktA, hydrogen peroxide

## Abstract

A psychrotolerant bacterium, strain T-3 (identified as *Psychrobacter piscatorii*), that exhibited an extraordinarily high catalase activity was isolated from the drain pool of a plant that uses H_2_O_2_ as a bleaching agent. Its cell extract exhibited a catalase activity (19,700 U·mg protein^−1^) that was higher than that of *Micrococcus luteus* used for industrial catalase production. Catalase was approximately 10% of the total proteins in the cell extract of the strain. The catalase (PktA) was purified homogeneously by only two purification steps, anion exchange and hydrophobic chromatographies. The purified catalase exhibited higher catalytic efficiency and higher sensitivity of activity at high temperatures than *M. luteus* catalase. The deduced amino acid sequence showed the highest homology with catalase of *Psycrobacter cryohalolentis*, a psychrotolelant bacterium obtained from Siberian permafrost. These findings suggest that the characteristics of the PktA molecule reflected the taxonomic relationship of the isolate as well as the environmental conditions (low temperatures and high concentrations of H_2_O_2_) under which the bacterium survives. Strain T-3 efficiently produces a catalase (PktA) at a higher rate than *Exiguobacterium oxidotolerans*, which produces a very strong activity of catalase (EktA) at a moderate rate, in order to adapt to high concentration of H_2_O_2_.

## 1. Introduction

Aerobic microorganisms encounter intracellular reactive oxygen species (ROS), such as superoxide (O_2_^•−^), hydrogen peroxide (H_2_O_2_) and hydroxyl radical (OH^•^), as byproducts of their own oxygen metabolism. H_2_O_2_ is produced from the reaction catalyzed by superoxide dismutase in O_2_^•−^ as the substrate [1]. The presence of H_2_O_2_ in microbial cells may lead to the generation of more harmful ROS, OH^•^, through the Fenton reaction [2]. Thus, elimination of intracellular H_2_O_2_ is crucial for microorganisms to prevent ROS reaction with their cell components such as DNA, RNA, proteins and lipids [2–4]. Furthermore, elimination of extracellular H_2_O_2_ is also important for not only aerobes but also anaerobes to survive in their own niches. Pathogenic, parasitic or symbiotic microorganisms interact with H_2_O_2_ produced by their host cells [5–7]. For example, *Vibrio fisheri* eliminates H_2_O_2_ produced by the host squid in order to survive in the light organ of the host [7]. Thus, elimination of H_2_O_2_ is very important for microorganisms not only intracellularly but also extracelluarly for sustaining their life and survival. In most cases, catalase production is involved as a defense mechanism.

The dismutation reaction of H_2_O_2_ in microorganisms has evolved in three phylogenetically unrelated protein types: monofunctional catalase, catalase-peroxidase and Mn-catalase [8,9]. Phylogenetic analysis based on the amino acid sequence of the monofunctioal catalase has revealed their subdivision into three distinct clades [10,11]. This phylogenetic tree is unrelated to that of the 16S rRNA gene sequence. Clade 1 catalases are small-subunit catalases are predominantly of plant origin but also non-pathogenic or opportunistic bacteria that are widespread in nature such as *Pseudomonas* species clade 2 catalases are large-subunit catalases including enzymes from bacteria and fungi. Clade 3 catalases are small-subunit catalases and they are derived from bacteria, archaea, fungi and eukaryotes. The bacteria possessing a clade 3 catalase and having restricted environment contain only a single catalase isozyme (e.g., *Haemophilus influenzae* and *Neisseria gonorrhoeae*).

There have been few reports on microorganisms that can survive in highly oxidative environments. Therefore, we have initiated the study on the bacteria adapted to the oxidative environment and their catalases. Bacterial strains have been isolated from a drain pool of a fish processing plant that uses H_2_O_2_ as a bleaching agent for brightening the color of herring egg. VktA catalase was first purified from a novel species, *Vibrio rumoiensis* S-1^T^ [12–15], which was isolated from the drain, and it exhibits high catalase activity and low heat resistance and belongs to clade 3 catalase [16,17]. We have also isolated a novel species, *Exiguobacterium oxidotolerans* T-2-2^T^ [18,19], from upstream of the drain of the same plant where strain S-1^T^ was isolated. The upstream contains a higher concentration of H_2_O_2_ than downstream. The catalase of *E. oxidotolerans* (EKtA) exhibits higher reactivity with larger substrates such as methyl hyroperoxide and peracetic acid owing to its wide bottleneck in the main channel [20]. EKTA is a clade 1 catalase. We have isolated the third strain, *Psychrobacter piscatorii* T-3-2^T^, whose cell extract exhibits 12,000 U·mg protein^−1^, from the same place where strain T-2-2^T^ was isolated [21]. In addition to strain T-3-2^T^, we have isolated *P. piscatorii* strain T-3 found to have similar characteristics to strain T-3-2^T^, which exhibited a higher activity than strain T-3-2^T^. Although the strain T-3 catalase expression system has been constructed in *Escherichia coli* [22], characterization of the strain and the biochemical characteristics of the catalase have not been reported. Therefore, characterization of strain T-3 and detailed biochemical characterization of the catalase are presented in this study.

## 2. Results and Discussion

### 2.1. Bacterial Identification

The 1510 bp sequence of the 16S rRNA gene of the isolate (strain T-3) was determined and its similarities with these in the database were estimated using the GENETYX ver. 10 computer program (Genetyx, Tokyo, Japan). The isolate showed the highest similarity with *P. piscatorii* T-3-2^T^ (99.9%). The phenotypic characteristics of the isolate were also similar to those of *P. piscatorii* T-3-2^T^ (data not shown). The DNA G + C mol% was 44.6%. On the basis of the above results, DNA-DNA relatedness between the isolate and *P. piscatorii* T-3-2^T^ was estimated. Taking all the findings together, the isolate was identified as *P. piscatorii* (100% DNA-DNA relatedness). The activity of the catalase of isolate was approximately 2 times higher than that of strain T-3-2^T^ under the same cell preparation conditions. Therefore, the isolate was used for further studies on catalase.

### 2.2. Purification of *P. piscatorii* T-3 Catalase

The purification steps for catalase from cell extract are summarized in Table 1. The cell extract exhibited a higher catalase activity (19,700 U·mg protein^−1^) than that of *Micrococcus luteus* (5000–10,900 U·mg protein^−1^) used for industrial catalase production [12,18,23]. The catalase was purified by one-step anion-exchange chromatography and one-step hydrophobic chromatography. The procedure does not require a gel filtration step, and this means that large amounts of starting materials (*i.e.*, cell extract) can be applied for the purification process. This procedure demonstrated approximately 11-fold purification with 22% yield. The purified catalase showed a final specific activity of 222,000 U·mg protein^−1^. A resting absorption ratio of 408 nm to 280 nm was 0.72.

### 2.3. Molecular Mass and Spectroscopic Properties of the Catalase

**Figure 1:**
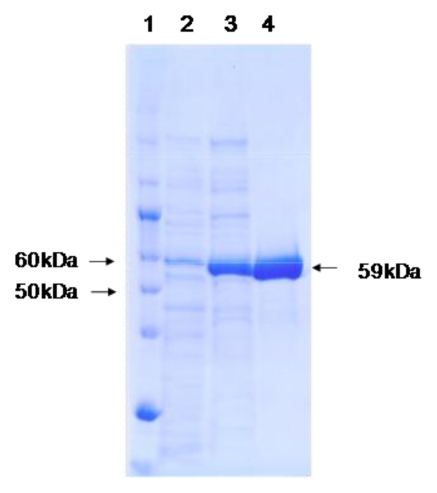
SDS-PAGE in each purification step for PktA from *Psychrobacter piscatorii* Lane 1, Protein marker; Lane 2, Crude extract; Lane 3, DEAE-Toyopearl 650M; Lane 4, phenyl Sepharose high performance.

The molecular mass of a subunit of catalase was estimated to be 59 kDa by SDS-PAGE (Figure 1). The native molecular mass was estimated to be 247 kDa by gel filtration. These findings suggest that the purified catalase is composed of four identical subunits. The absorption spectrum of purified catalase exhibited a Soret band at 406 nm and additional minor peaks between 500–550 nm and 630 nm (data not shown).

### 2.4. Enzymatic Characterization of the Catalase

The kinetic analysis of catalase (PktA) activity showed an apparent *V*_max_ of 2.35 × 10^5^ μmol H_2_O_2_·μmol heme^−1^·s^−1^ and an apparent *K*_m_ of 75 mM for H_2_O_2_ (Table 2). The catalytic efficiency, *V*_max_/*K*_m_, of the catalase from the isolate was higher than that from *M. luteus*. The *V*_max_/*K*_m_ of PktA was higher than those of several clade 3 catalases from pathogenic or symbiotic bacteria except for *Xanthomonas campestris* catalase [24]. This catalytic property may protect the bacterium in the presence of high concentrations of H_2_O_2_ in the original environment from which the strain was isolated.

**Figure 2:**
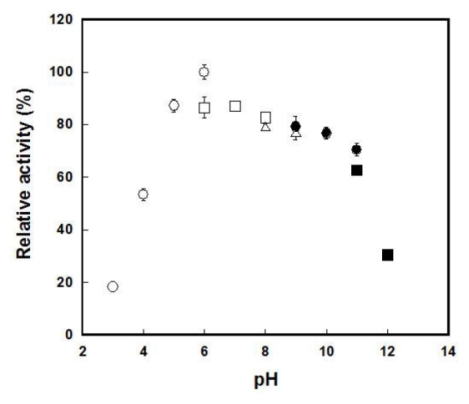
Effect of pH on activity of PktA. The buffers (50 mM) used were as follows: pHs 3.0–6.0, sodium citrate (open circles); pHs 6.0–8.0, sodium phosphate (open squares); pHs 8.0–9.0, Tri-HCl (open triangles); pHs 9.0–11.0, Na_2_HCO_3_-NaOH (closed circles); pHs 11.0–12.0, NaHPO_4_-NaOH (closed squares).

The pH dependence of the PktA activity was studied in a pH range from 3.0 to 12.0. A broad optimum pH range was observed from pH 5.0 to pH 10.0. Residual activity was observed even at pH 4.0 (53.4%) or pH 3 (18.4%) (Figure 2). On the other hand, the *M. luteus* catalase showed relative activities of 23.5% and 0.03% at pH 4.0 and pH 3.0, respectively (data not shown). The pH stability of PktA activity was estimated by incubating in a buffer solution (pH range from 3.0 to 11.0) at 60 °C for 15 min. The enzyme was most stable at pH 8.0 (data not shown). The activity of this enzyme was completely eliminated at pH 4.5 and pH 11.

**Figure 3:**
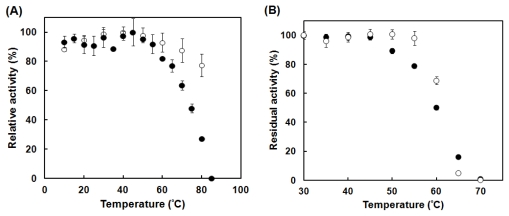
Effects of temperature on catalase activity (**A**) and stability (**B**) of PktA and *M. luteus* catalase. (**A**) Catalase activity was assayed, as described in Experimental Section, at temperatures indicated. (**B**) The enzymes were incubated for 15 min at the indicated temperatures prior to activity estimation. Catalase activity was assayed as described in Experimental Section at 25 °C. Symbols: PktA (closed circles); *M. luteus* catalase (open circles).

PktA activity was estimated at various temperatures and was compared with *M. luteus* catalase activity (Figure 3A). Although the temperature dependence of *M. luteus* catalase was scarcely observed, PktA exhibited a clear decrease in its activity at temperature higher than 55 °C. On the other hand, the decrease in activity with the decrease in temperature was scarcely observed in both PktA and *M. luteus* catalases. The stability of catalase activity depending on temperature was determined by incubation of the enzyme at temperatures ranging from 30 °C to 70 °C for 15 min (Figure 3B). Both PktA and *M. luteus* catalase are stable up to 45 °C. Although PktA started to lose its activity at 50 °C, *M. luteus* catalase was stable until 55 °C. Although the residual activity of *M. luteus* catalase was higher at 50–60 °C than that of PktA, the former was lower than the latter at 65 °C.

**Figure 4:**
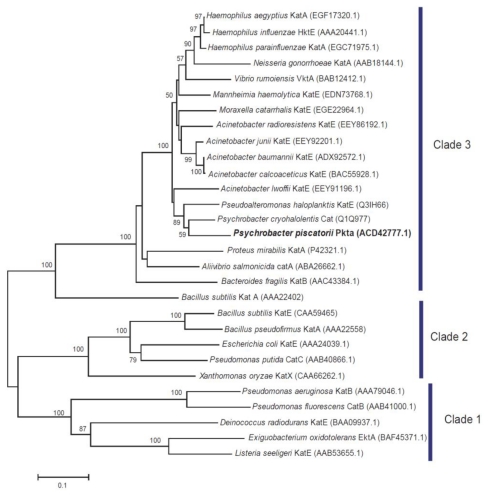
Phylogenetic position of PktA among bacterial catalases. The tree was constructed using the CLUSTAL W program [25] with multiple alignments using the neighbor-joining method [26]. Numbers at the branches are bootstrap percentages based on 1000 replicates. Bar, 0.1 changes per amino acid position. The amino acid sequences of catalases used for the alignment are shown with their GenBank/DDBJ/EMBL accession numbers in parentheses.

The entire 1533 bp DNA sequence that includes the entire nucleotide sequence of PktA was determined. The deduced amino acid sequence showed that PktA is composed of 510 amino acids with a calculated size of 58,313.19 Da. The deduced amino acid sequence showed the highest identity (84.9%) with catalase of *Psycrobacter cryohalolentis*. A comparison of the deduced catalase amino acid sequence revealed that the active sites (His^65^, Ser^104^ and Asn^138^), binding sites of the distal region of heme (Val^106^, Thr^128^ and Phe^143^) and proximal sites of heme (Tyr^348^ and Arg^355^) are well conserved. PktA may contain NADPH inside of its molecule because NADPH-binding sites (His^184^, Arg^193^, Val^292^ and Lys^295^) were observed [27]. The deduced amino acid sequence showed that PktA is a clade 3 catalase in the constructed phylogenetic tree (Figure 4). PktA showed the closest kinship with catalase of *P cryohalolentis*, a psychrotolelant bacterium obtained from Siberian permafrost [28]. Although it has been reported that the phylogenetic tree constructed on the basis of the amino acid sequence is not related to that constructed on the basis of the 16S rRNA gene sequence, PktA occupied a position related to that of the catalase from a taxonomic neighbor, *P. cryohalolentis*.

### 2.5. DNA Gene Sequence of the Catalase

To determine of the sequence of *PktA*, the N-terminal amino acid sequence was determined. The determined amino acid sequence including an unspecified amino acid, X, is: SNDMNDKKXPYDMTPLXMXN. On the basis of this amino acid sequence, degenerated mix primers were designed for amplification of the partial gene sequence of *PktA* by PCR. The approximately 1-kbp product was obtained and its gene sequence was determined. The obtained gene sequence showed higher than 80% similarity with the catalase gene sequence of *Psychrobacter cryohalolentis*. On the basis of the determined sequence and the gene sequence of catalase from *P. cryohalotentis*, two primer sets were designed and a further extended sequence was determined by gene walking in the fragment digested with *Hind* III and *Xba* I on the 5′ side and 3′ side, respectively with a Takara PCR *in vitro* Cloning kit (Takara Bio, Ohtsu, Siga, Japan).

## 2.6. Discussion

To determine the types of microorganism selected in an oxidative environment and the types of catalase molecule they produce, we examined such microorganisms and their catalases. *V. rumoiensis* S-1^T^ [12–15] and *E. oxidotolerans* T-2-2^T^ [18,19] were respectively isolated from downstream and upstream of the drain pool of a plant that uses H_2_O_2_ as a bleaching agent. The catalase activities of the crude extracts of the former and the latter are 7300 U·mg protein^−1^ [16] and 24,600 U·mg protein^−1^ [19], respectively. The higher catalase activity of the former is probably due to the fact that the upstream of the drain pool contains a higher concentration of H_2_O_2_ that the downstream. The catalase activity of the crude extract of *P. piscatorii* T-3 (19,700 U·mg protein^−1^), which was isolated from upstream, is also higher than that of *V. rumoiensis* S-1^T^ [16]. Catalase contained approximately 10% of the total proteins in the cell extract of strain T-3. In addition the enzyme was purified homogeneously using only two purification steps. The high catalase activity is due to not only the high content of catalase but also the low content of background proteins. On the other hand, purified catalase, PktA (222,000 U·mg protein^−1^), exhibited an activity that was not as high as those of *V. rumoiensis* S-1^T^ (395,000 U·mg protein^−1^) [16] and *E. oxidotolerans* T-2-2^T^ (430,000 U·mg protein^−1^) [20] catalases under the same assay conditions. The *V. rumoiensis* S-1^T^ and *E. oxidotolerans* T-2-2^T^ catalases produced are 1.8% and 6.5% of the cell extract, respectively. This suggests that the catalase molecules with the highest activity were not always selected from the environment containing high concentrations of H_2_O_2_. Strain T-3 efficiently produces a catalase (PktA) at a higher rate than *V. rumoiensis* S-1^T^ and *E. oxidotolerans* T-2-2^T^, which produce very strong catalases, VktA and EktA, respectively, at a moderate rates, in order to adapt to high concentration of H_2_O_2_.

PktA exhibited a relatively high catalytic efficiency. This characteristic may be related to the protection of microorganism against high concentrations of H_2_O_2_. Several symbiotic and pathogenic microorganisms possess clade 3 catalase as the sole catalase [10]. It is considered that some of the clade 3 catalases involved in symbiosis and pathogenesis have evolved for the protection of microorganisms from their hosts. Although *P. piscatorii* T-3 possesses the clade 2 catalase gene, it expresses only clade 3 catalase (data not shown). This suggests that the bacterium selects an effective catalase that is expressed to counteract high concentrations of H_2_O_2._

The temperature dependence profile between the temperature range of 10–70 °C of catalase activity has not been reported. Actually, bovine liver and *M. luteus* catalase activities do not exhibit a clear temperature dependence [16]. *V. rumoiensis* S-1^T^ catalase, VktA, is found as the first catalase showing temperature dependence in psychrotolerant microorganisms; it exhibits an optimum temperature of 40 °C. In 2006, *Aliivibrio salmonicida* (*Vibrio salmonicida*) catalase is found as the first psychrophilic catalase and it exhibits an optimum temperature range of 0–10 °C [29]. On the other hand, mesophile [30] and thermophile [30] catalases exhibit optimum temperatures of 60 °C and 70 °C, respectively. *E. oxidotolerans* T-2-2^T^ catalase also exhibited a temperature dependence profile and an optimum temperature of 45 °C (unpublished result). Although PktA did not exhibit a clear temperature dependence between 10 and 55 °C, it exhibited obvious temperature dependence at 60–85 °C. On the basis of above findings, it can be predicted that microorganisms living in a cold environment (lower than approximately 5 °C) possess temperature-dependent catalases. Therefore, it is considered that temperature-dependent catalase molecules are selected in cold environments.

The amino acid sequence of the catalase of the isolate showed the highest homology with the catalase of *P. cryohalolentis*, a psychrotolelant bacterium obtained from Siberian permafrost [28]. This finding suggests that the characteristics of the PktA molecule reflected the taxonomic relationship of the isolate. It is also suggested that certain taxonomic groups of bacteria have an ability to respond to several extreme environments [32].

## 3. Experimental Section

### 3.1. Chemicals and Enzyme

Standard chemicals were purchased from Wako Pure Chemicals unless otherwise stated. They were of the highest grade available and were used without further purification. *Micrococcus luteus* catalase was purchased from Nagase ChemteX and was purified by size exclusion chromatography (Sephacryl S-300 high resolution, 2.6 cm × 89 cm) and equilibrated with 50 mM potassium phosphate buffer (pH 7.0) containing 0.25 M NaCl.

### 3.2. Bacterial Strain

A drain water sample (its temperature was approximately 5 °C) was obtained from a fish egg processing plant in Rumoi, Hokkaido, in which H_2_O_2_ is used as a bleaching agent. An aliquot of a sample was plated onto a 10 mM H_2_O_2_-containing PYS-2 plate (pH 7.5) containing (per liter of deionized water) 8 g polypepton (Nihon Pharmaceuticals), 3 g yeast extract (Kyokuto) and 5 g NaCl, and incubated at 27 °C for 1 week. One white colony was picked up from the incubated plate and was identified as strain T-3.

### 3.3. Phenotypic Characterization of Strain T-3

PYS-2 medium, incubated at 27 °C, was used for the phenotypic characterization as the basal condition. Morphological, physiological and biochemical characterizations were performed as described by Barrow and Feltham [33]. Carbohydrate metabolism was examined by the method of Hugh and Leifson [34]. Alginase activity was determined after an inoculated agar plate was overlaid with ethanol after 10 days of cultivation.

### 3.4. 16 rRNA Sequencing

DNA was extracted using InstaGen™ Matrix (Bio-Rad) according to the manufacturer’s instruction. The 16S rRNA gene was amplified by PCR using universal primers 9F (5′-GAGTTTGATCCTGGCTCAG-3′) and 1541R (5′-AAGGAGGTGATCCAGCC-3′). The resulting PCR product was purified using a QIAquick PCR purification kit (Qiagen) and sequenced directly by the dideoxynucleotide chain-termination method using a DNA sequencer (PRISM 3100; Applied Biosystems) with a BigDye Termination RR mix version 3.1 (Applied Biosystems) according to the manufacturer’s instruction. The determined 16S rRNA gene sequence of strain T-3 has been deposited in GenBank/DDBJ/EMBL under the accession number AB688097. Multiple alignments of the sequences were performed using the clustal w program [25]. A phylogenetic tree was constructed by the neighbor-joining [26] method using MEGA 5 [35]. For neighbour-joining analysis, the distance between sequences (*K*_unc_) was calculated using Kimura’s two-parameter model [36]. The confidence values for the branches of the phylogenetic tree were determined using bootstrap analysis [37] based on 1000 resamplings. The similarity between sequences was calculated using the genetyx ver. 10 computer program (Genetyx, Tokyo, Japan).

### 3.5. DNA Base Composition and DNA-DNA Hybridization

DNA was prepared from bacterial cells by the method of Marmur [38]. The DNA obtained was digested with nuclease P1 (Yamasa Shoyu) and the resulting nucleotides were separated by HPLC [39].

The level of DNA-DNA relatedness was determined fluorometrically by the method of Ezaki *et al*. [40] using photobiotin-labelled DNA probes and black microplates.

### 3.6. Enzyme Assay Condition

Catalase activity was measured spectrophotometrically by monitoring the initial decrease in absorbance at 240 nm caused by the disappearance of H_2_O_2_, using a spectrophotometer (Hitachi U-3210) at 25 °C. The concentration of H_2_O_2_ was determined on the basis of the extinction coefficient of 43.6 M^−1^·cm^−1^ [41]. The standard reaction mixture for the assay contained 50 mM potassium phosphate buffer (pH 7.0), 30 mM H_2_O_2_ and 10 μL of a catalase solution in a total volume of 1.0 mL. The amount of enzyme activity that decomposed 1 μmol of H_2_O_2_ per min was defined as 1 U. The enzyme activities are expressed as the means of at least four times measurements.

The initial absorbance attributed to H_2_O_2_ as determined by spectrophotometry is not accurate in the presence of high concentrations of H_2_O_2_ neither is H_2_O_2_ in an alkaline solution. Catalase activity was determined using a galvanic-type oxygen electrode (Iijima Electronics Corporation, Aichi, Japan) for the determination of kinetic parameter measurement and pH dependence by the measurement of production of O_2_ produced by the catalytic reaction at 25 °C by the method of Rørth and Jensen [42]. Initial rates of oxygen production were used to determine the activity. The standard reaction mixture for the assay contained 50 mM potassium phosphate buffer (pH 7.0), 30 mM H_2_O_2_ and 10 μL of a catalase solution in a total volume of 1.6 mL. The amount of enzyme activity that decomposed 1 μmol of H_2_O_2_ per min was defined as 1 U.

### 3.7. Purification of Catalase from Strain T-3(PktA)

Strain T-3 was cultivated aerobically up to the early stationary growth phase at 27 °C in PYS-3 medium (pH 7.5) containing (per liter of deionized water) 8 g polypepton (Nihon Pharmaceuticals, Tokyo, Japan), 3 g yeast extract (Kyokuto, Tokyo, Japan), 5 g NaCl, 5 g sodium succinate. The organism was cultured in 500 mL of above-mentioned medium in a 2 L baffled flask (×4) set on a rotary shaker (120 rpm). Cells were harvested by centrifugation at 7000 g for 20 min and frozen at −30 °C until use.

Frozen cells were suspended in buffer A (three times weight of the cells) consisting of 10 mM Tris-HCl (pH 8.3) and 1 mM EDTA (2·Na). Then, 1 μg·mL^−1^ of 2000 U of DNase I (Sigma, St. Louis, Mo, USA) and 7 mM MgCl_2_·6H_2_O were added to the suspension and the suspension was passed through a French pressure cell (SLM-AMINCO Instrument, Rochester, NY, USA) at 105 kgf/cm^2^. The resulting fluid was centrifuged at 13,000 g to remove unbroken cells, dialyzed against buffer A and was applied onto a DEAE-Toyopearl 650M column (Tosoh, Tokyo, Japan; 2.5 cm × 10 cm) equilibrated with buffer A. The column was washed with 10 column volume of buffer A and was eluted with a linear gradient of 125–250 mM NaCl containing buffer A. The active fractions were collected and solid ammonium sulfate was added to the enzyme solution at a final concentration of 1 M. The enzyme solution was applied to a phenyl-Sepharose high-performance column (GE Healthcare Life Sciences Buckinghamshire, UK) column (1.6 cm × 10 cm) equilibrated with buffer A containing 1 M ammonium sulfate. The enzyme was eluted with a linear gradient of 1–0.4 M ammonium sulfate in buffer A. The eluted active fractions were collected and dialyzed against buffer A and then concentrated by ultrafiltration using an Amicon YM-30 membrane (Amicon Inc., Beverly, MA, USA).

### 3.8. Physical and Chemical Measurements

Spectrophotometry was performed using a Cary 100 UV-Vis spectrophotometer (Varian, Palo Alto, CA, USA) using a 1-cm-light-path cuvette. The molecular mass of catalase was determined by SDS-PAGE using 10% (w/v) acrylamide gel (c-PAGEL, Atto, Tokyo, Japan) according to the method of Laemmli and Favre [43]. The gel was stained with Coomassie Brilliant Blue R-250 (CBB). The molecular weight of the native enzyme was determined by gel filtration using two 7.8 mm × 300 mm Protein PAK 300 columns (Nihon Waters, Tokyo, Japan) equilibrated with 0.1 M potassium phosphate buffer (pH 7.0). For molecular mass standards, the following proteins were used: thyroglobulin (669 kDa), apoferritin (443 kDa), α-amylase (200 kDa), alcohol dehydrogenase (150 kDa), bovine serum albumin (66.2 kDa) and carbonic anhydrase (29 kDa).

### 3.9. Protein Sequencing

Purification products separated by SDS-PAGE, as performed as described in the above section, were transferred to a polyvinylidene fluoride (PVDF) membrane using a semidry blotter (AE-6677G Holize Blot). The band corresponding to catalase was cut off from the membrane and applied to a protein sequencer (Model 491, Perkin-Elmer, Winter Street Waltham, MA, USA) to determine the N-terminal amino acid sequences of the polypeptide by Edman degradation [44]. The N-terminal amino acid sequence of the catalase from the isolate was similar to the catalases from *Psychrobacter* species.

### 3.10. Determination of Gene Sequence of PktA Catalase

Strain T-3 was cultured in PYS-3 broth as described above for 24 h, and DNA was extracted using InstaGen™ Matrix (Bio-Rad) according to the manufacturer’s instruction. The extracted DNA was used as a template for PCR amplification. The degenerated mix primers 5′-AAYGAYATGAAYGAYAARAA-3′ and 5′-TGNGCRTCNGCRTARTTRAA-3′ were designed on the basis of the determined N-amino acid sequence of PKTA and amino acid sequences of highly conserved regions among catalase from five strains belonging to the genus *Psychrobacter*, respectively. The amplified PCR product of approximately 1 kb was obtained and its gene sequence was determined. On the basis of the determined sequence, a further extended sequence was determined by gene walking using a Takara PCR *in vitro* Cloning kit (Takara Bio, Ohtsu, Siga, Japan). The DNA gene sequence was determined by the dideoxynucleotide chain-termination method using an Applied Biosystems PRISM 3100 DNA sequencer (Perkin-Elmer, Wellesley, MA, USA) with a BigDye Termination RR mix version 3.1 (Perkin-Elmer) according to the manufacturer’s instruction. The PktA sequence has been deposited in the Genbank/DDBJ/EMBL under the accession number EU543218. Multiple alignments, construction of phylogenetic tree and analysis of gene sequence similarity or identity were performed as described in the *16 rRNA Sequencing* section.

## 4. Conclusions

The catalase, PktA, of *P. piscatorii* T-3 was a dominant protein (10%) in the cell extract, which contained a relatively small amount of background proteins. The purified catalase exhibited higher catalytic efficiency, higher sensitivity of its activity at high temperatures and higher relative activity at pHs 3 and 4 than *M. luteus* catalase, which is an industrial enzyme. The amino acid sequence of PktA deduced from its gene sequence showed that the enzyme is a clade 3 catalase and shows the highest homology with catalase of *P cryohalolentis*, a psychrotolerant bacterium obtained from Siberian permafrost. These findings suggest that the characteristics of the PktA molecule reflected the taxonomic relationship of the isolate as well as the environmental conditions (low temperatures and high concentrations of H_2_O_2_) under which the bacterium survives. Strain T-3 produces a catalase (PktA) at a higher rate than *E. oxidototolerans* T-2-2^T^, which produces a very strong catalase (EktA), at a moderate rate, in order to adapt to a high concentration of H_2_O_2_.

## Figures and Tables

**Table 1:** Purification of PktA.

Step	Total protein (mg)	Total activity (U × 10^3^)	Specific activity (U·mg^−1^)	Purification (fold)	Yield (%)
Crude extract	1130	22,300	19,700	1.0	100
DEAE-Toyopearl	120	17,000	142,000	7.2	76
Phenyl Sepharose	22.2	4940	222,000	11.0	22

**Table 2:** Apparent kinetic parameters of clade 3 catalases.

Source	*V*_max_^a^	*K*_m_ (mM)	*V*_max_/*K*_m_
*Psychrobacter piscatorii* T-3	235,000	75	3133
*Micrococcus luteus*	284,000	147	1931
*Bacteroides fragilis*	241,000	128	1883
*Helicobacter pylori*	250,000	108	2315
*Serratia marcescens*	228,000	180	1267
*Xanthomonas campestris*	244,000	64	3812

a *V*_max_ is in μmol H_2_O_2_·μmol heme^−1^·s^−1^; Data of other bacteria are cited from the report by Switala and Loewen [24].

## References

[1] Bolton J.L., Trush M.A., Penning T.M., Dryhurst G., Monks T.J. (2000). Role of quinones in toxicology. Chem. Res. Toxicol.

[2] Halliwell B., Gutteridge J.M.C. (1999). Free Radical in Biology and Medicine.

[3] Imlay J.A., Linn S. (1988). DNA damage and oxygen radical toxicity. Science.

[4] Rowe L.A., Degtyareva N, Doetsch P.W. (2008). DNA damage-induced reactive oxygen species (ROS) stress response in *Saccharomyces cerevisiae*. Free Radic. Biol. Med..

[5] Katsuwon J., Anderson A.J. (1992). Characterization of catalase activities in root colonizing isolates of *Pseudomonas putida*. Can. J. Microbiol.

[6] Rocha E.R., Selby T., Coleman J.P., Smith C.J. (1996). Oxidative stress response in an anaerobe, *Bacteroides fragilis*: A role for catalase in protection against hydrogen peroxide. J. Bacteriol.

[7] Visick K.L., Ruby E.G. (1998). The periplasmic, group III catalase *Vibrio fisheri* is required for normal symbiotic competence and is induced both by oxidative stress and by approach to stationary phase. J. Bacteriol.

[8] Loewen P.C., Klotz M.G., Hassett D.J. (2000). Catalase—an “old” enzyme that continues to surprise us. ASM News.

[9] Zamocky M., Furtmüller P.G., Obinger C. (2008). Evolution of catalases from bacteria to humans. Antioxid. Redox Signal.

[10] Klotz M.G., Klassen G.R., Loewen P.C. (1997). Phylogenetic relationships among prokaryotic and eukaryotic catalases. Mol. Biol. Evol.

[11] Klotz M.G., Loewen P.C. (2003). The molecular evolution of catalase hydroperoxidase: Evidence for multiple lateral transfer of gene between prokaryota and from bacteria into eukaryote. Mol. Biol. Evol.

[12] Yumoto I., Yamazaki K., Kawasaki K., Ichise N., Morita N., Hoshino T., Okuyama H. (1998). Isolation of *Vibrio* sp. S-1 exhibiting extraordinarily high catalase activity. J. Ferment. Bioeng.

[13] Yumoto I., Iwata H., Sawabe T., Ueno K., Ichise N., Matsuyama H., Okuyama H., Kawasaki K. (1999). Characterization of a facultatively psychrophilic bacterium, *Vibrio rumoiensis* sp. nov., that exhibits high catalase activity. Appl. Environ. Microbiol.

[14] Ichise N., Morita N., Hoshino T., Kawasaki K., Yumoto I., Okuyama H. (1999). A mechanism of resistance to hydrogen peroxide in *Vibrio rumoiensis* S-1. Appl. Environ. Microbiol.

[15] Ichise N., Hirota K., Ichihashi D., Nodasaka Y., Morita N., Okuyama H., Yumoto I. (2008). H2O2 tolerance of *Vibrio rumoiensis* S-1T is attributable to the cellular catalase activity. J. Biosci. Bioeng.

[16] Yumoto I., Ichihashi D., Iwata H., Istokovics A., Ichise N., Matsuyama H., Okuyama H., Kawasaki K. (2000). Purification and characterization of a catalase from the facultative psychrophilic bacterium *Vibrio rumoiensis* S-1T exhibiting high catalase activity. J. Bacteriol.

[17] Ichise N., Morita N., Kawasaki K., Yumoto I., Okuyama H. (2000). Gene cloning and expression of the catalase from the hydrogen peroxide-resistant bacterium *Vibrio rumoiensis* S-1 and its subcellular localization. J. Biosci. Bioeng.

[18] Yumoto I., Hishinuma-Narisawa M., Hirota K., Shingyo T., Takebe F., Nodasaka Y., Matsuyama H., Hara I. (2004). *Exiguobacterium oxidotolerans* sp. nov., a novel alkaliphile exhibiting high catalase activity. Int. J. Syst. Evol. Microbiol.

[19] Takebe F., Hara I., Matsuyama H., Yumoto I. (2007). Effect of H2O2 under low- and high-aeration-level conditions on growth and catalase activity in *Exiguobacterium oxidotolerans* T-2-2T. J. Biosci. Bioeng.

[20] Hara I., Ichise N., Kojima K., Kondo H., Ohgiya S., Matsuyama H., Yumoto I. (2007). Relationship between the size of the bottleneck 15 Å away from iron in the main channel and reactivity of catalase corresponding to the molecular size of substrates. Biochemistry.

[21] Yumoto I., Hirota K., Kimoto H., Nodasaka Y., Matsuyama H., Yoshimune K. (2010). *Psychrobacter piscatorii* sp. nov., a psychrotolerant bacterium exhibiting high catalase activity isolated from an oxidative environment. Int. J. Syst. Evol. Microbiol.

[22] Kimoto H., Matsuyama H., Yumoto I., Yoshimune K. (2008). Heme content of recombinant catalase from *Psychrobacter* sp. T-3 altered by host *Escherichia coli* growth conditions. Protein Expr. Purif.

[23] Nakayama M., Nakajima-Kambe T., Katayama H., Higuchi K., Kawasaki Y., Fujii R. (2008). High catalase production by *Rhizobium radiobacter* strain 2-1. J. Biosci. Bioeng.

[24] Switala J., Loewen P.C. (2002). Diversity of properties among catalases. Arch. Biochem. Biophys.

[25] Thompson J.D., Higgins D.G., Gibson T.J. (1994). CLUSTAL W: Improving the sensitivity of progressive multiple sequence alignment through sequence weighting, position-specific gap penalties and weight matrix choice. Nucleic Acid Res.

[26] Saitou N., Nei M. (1987). The neighbor-joining method: A new method for reconstructing phylogenetic trees. Mol. Biol. Evol.

[27] Zámocký M., Koller F. (1999). Understanding the structure and function of catalases: Clues from molecular evolution and in vitro mutagenesis. Prog. Biophys. Mol. Biol.

[28] Bakermans C., Ayala-del-Río H.L., Ponder M.A., Vishnivetskaya T., Gilichinsky D., Thomashow M.F., Tiedje J.M. (2006). *Psychrobacter cryohalolentis* sp. nov. and *Psychrobacter arcticus* sp. nov., isolated from Siberian permafrost. Int. J. Syst. Evol. Microbiol.

[29] Loewntzen M.S., Moe E., Jouve H.M., Willassen N.P. (2006). Cold adapted features of *Vibrio salmonicida* catalase: characterization and comparison to the mesophilic counterpart from *Proteus mirabilis*. Extremophiles.

[30] Yamaguchi H., Sugiyama K., Hosoya M., Takahashi S., Nakayama T. (2011). Gene cloning and biochemical characterization of a catalase from *Gluconobacter oxydans*. J. Biosci. Bioeng.

[31] Wang H., Tokushige Y., Shinoyama H., Fujii T., Urakami T. (1998). Purification and characterization of thermostable catalase from culture broth of *Thermoascus aurantiacus*. J. Ferment. Bioeng.

[32] Rodrigues D.F., da C Jesus E., Ayala-Del-Río H.L., Pellizari V.H., Gilichinsky D., Sepulveda-Torres L., Tiedje J.M. (2009). Biogeography of two cold-adapted genera: *Psychrobacter* and *Exiguobacterium*. ISME J.

[33] Barrow G.I., Feltham R.K.A. (1993). Cowan and Steel’s Manual for the Identification of Medical Bacteria.

[34] Hugh R., Leifson E. (1953). The taxonomic significance of fermentative versus oxidative metabolism of carbohydrates by various gram negative bacteria. J. Bacteriol.

[35] Tamura K., Peterson D., Peterson N., Stecher G., Nei M., Kumar S. (2011). MEGA5: Molecular evolutionary genetic analysis using maximum likelihood, evolutionary distance, and maximum parsimony methods. Mol. Biol. Evol.

[36] Kimura M. (1980). A simple method for estimating evolutionary rates of base substitutions through comparative studies of nucleotide sequences. J. Mol. Evol.

[37] Felsenstein J. (1981). Evolutionary trees from DNA sequences: A maximum likelihood approach. J. Mol. Evol.

[38] Marmur J. (1961). A procedure for the isolation of deoxyribonucleic acid from micro-organismss. J. Mol. Biol.

[39] Tamaoka J., Komagata K. (1984). Determination of DNA base composition by reversed-phase high-performance liquid chromatography. FEMS Microbiol. Lett.

[40] Esaki T., Hashimoto Y., Yabuuchi E. (1989). Fluorometric deoxyribonucleic acid–deoxyribonucleic acid hybridization in micro-dilution wells as an alternative to membrane filter hybridization in which radioisotopes are used to determine genetic relatedness among bacterial strains. Int. J. Syst. Bacteriol.

[41] Hildebraunt A.G., Roots I. (1975). Reduced nicotinamide adenine phosphate (NADH)-dependent formation and breakdown of hydrogen peroxide during mixed function oxidation reactions in liver microsomes. Arch. Biochem. Biophys.

[42] RØrth M., Jensen P.K. (1967). Determination of catalase activity by means of the Clark oxygen electrode. Biochem. Biophys. Acta.

[43] Laemmli U.K., Favre M. (1973). Maturation of the head of bacteriophage T4. I. DNA packaging events. J. Mol. Biol.

[44] Edman P., Henschen A, Needlman S.B. (1975). Sequence Determination. Protein sequence determination.

